# Strong ion gap can be accurately estimated with a simple bedside equation

**DOI:** 10.1186/cc12382

**Published:** 2013-03-19

**Authors:** L Busse, L Chawla, R Panchamia, D Choi, E Nobakht, E Brasha-Mitchell, M Seneff

**Affiliations:** 1 George Washington University Medical Center, Washington, DC, USA

## Introduction

The anion gap (AG) is used routinely in the assessment of metabolic acidosis, but can be misleading in patients with hypoalbuminemia and other disorders commonly encountered in intensive care. This approach to acid-base analysis relies on assessment of pH, pCO_2_, sodium, bicarbonate and chloride, and can lead to underestimation or overestimation of the true electrochemical status of a patient, as it does not include important ions such as lactate, calcium, magnesium, and albumin. The strong ion gap (SIG) is an alternative to the AG and is based upon Stewart's physical chemistry approach. However, the SIG is cumbersome to calculate. As such, a number of shortcut equations have been developed in an effort to approximate the SIG. We sought to compare three such equations, the Kellum corrected anion gap (KellAGc), the Moviat equation, and EZSIG, in an effort to evaluate precision and accuracy [1-3].

## Methods

We conducted a retrospective chart review of consecutive patients admitted to the ICU of George Washington University Medical Center from September 2010 to March 2011. Of the 1,516 patients screened, 200 met inclusion criteria, which included availability of all laboratory components to calculate the SIG, obtained within 1 hour of each other. Demographic data and serum values for pH, pCO_2_, albumin, lactate, sodium, potassium, chloride, bicarbonate, magnesium, phosphate, and calcium were collected. The AG, SIG, KellAGc, EZSIG, and Moviat equations were subsequently calculated and compared using Pearson correlation and Bland-Altman analysis.

## Results

The mean SIG was 3.25 ± 3.5. Mean values for KellAGc, Moviat, and EZSIG were 4.5 ± 5.0, 1.77 ± 2.2, and 3.6 ± 3.7, respectively. Pearson correlation coefficients for KellAGc, Moviat, and EZSIG when compared with the SIG were *r *= 0.77, *P *= 0.0001; *r *= 0.88, *P *= 0.001; and *r *= 0.89, *P *= 0.001, respectively. In Bland-Altman analysis, the mean bias for the test equations versus the SIG were: KellAGc (1.25), Moviat (-1.48), and EZSIG (0.40).

## Conclusion

While all three equations correlated highly with the SIG, the EZSIG and Moviat outperformed the KellAGc in Pearson and Bland-Altman analysis. The EZSIG had a smaller bias than the Moviat equation and a slightly better correlation (0.89 vs. 0.88). In the assessment of critically ill patients, EZSIG is a candidate scanning equation for the measurement of the SIG when all SIG components are not available.
